# Silicon–calcium fertilizer increased rice yield and quality by improving soil health

**DOI:** 10.1038/s41598-024-63737-x

**Published:** 2024-06-07

**Authors:** Shuai Yuan, Yu Han, Can Cui, Pingping Chen, Naimei Tu, Zhongwen Rang, Zhenxie Yi

**Affiliations:** College of Agronomy, Hunan Agricultural University, Changsha, 410128 China

**Keywords:** Rice, Silicon–calcium fertilizer, Rice quality, Soil characteristics, Plant biotechnology, Plant physiology

## Abstract

It is important to ensure the nutritional quality and safe production of rice. Here, plot experiments were used to analyze the effects of three soil amendments—10 t ha^−1^ of biochar (BC), 1.5 t ha^−1^ of lime (LM), and 2.25 t ha^−1^ of silicon–calcium fertilizer (SC)—on the soil characteristics, rice yield and quality of double-cropping rice grown in mildly cadmium-polluted paddy fields. Compared with the control treatment (CK), the BC and SC treatments significantly improved rice processing, appearance and nutritional quality, but reduced cooking quality. All three soil amendments significantly reduced cadmium (Cd) content in brown rice. Soil amendments could significantly increase soil pH and reduce soil available Cd content. The application of the BC and SC treatments increased the content of each nutrient index in the soil (SOM, NN, AP, AK). Correlation analysis showed that the improvement in rice processing, appearance, and nutritional quality was mainly affected by the comprehensive effects of soil SOM, NN, AP and AK; the hygiene quality was mainly affected by soil pH and available Cd. In terms of benefit analysis combined with cost, the SC treatment had the highest benefit effect. Taken together, in mildly cadmium-polluted paddy fields, the application of silicon–calcium fertilizer improved the soil quality, thereby increased the yield and quality of rice, and had the best effect on increasing income.

## Introduction

Soil heavy metal pollution poses a substantial threat to food safety production and human health. The heavy metal pollution situation in China's farmland soil is severe. The exceedance rate of farmland soil points is 19.4%, with cadmium (Cd) pollution being the most prominent^1^. In the soil, Cd has high mobility, is easily absorbed by crops and can enter the food chain through the enrichment of agricultural products. The long-term intake of heavy metals can cause varying degrees of harm to the skin, bones, nervous system and internal organs of the human body^2^. Studies have shown that a variety of foods including grains are important features of a balanced diet, and it is necessary to ensure that the daily intake of grains is not less than 50–100 g^3^. Rice is one of the main food crops for people. Its own characteristics and planting methods allow it to easily absorb and enrich heavy metals in the environment, which poses a threat to the quality and safety of rice. With the continuous improvement in living standards, people's consumption demand for agricultural products has shifted from quantity to quality. Therefore, it is important to ensure both the safety and nutritional quality of rice production.

In-situ passivation restoration technology involves adding a soil amendment agent to the soil to improve soil quality by changing the physical and chemical properties such as soil pH^4,5^. This approach is widely used in soil remediation and treatment because of it has the advantages of good effect, simple operation, and easy to be promoted and used in large areas in farmland with light to moderate heavy metal pollution. Although passivating agents, including lime, biochar, clay minerals, fertilizers and some other soil amendments, can significantly reduce the availability of heavy metals in soil and their accumulation in plants, the existing disadvantages^6,7^, such as high production costs and the degree of applicability in production practice, cannot be ignored.

Two of the most useful soil amendment elements for rice are silicon and calcium. Silicon is considered a beneficial element for the healthy growth and development of crops. Rice is a typical silicon-loving plant and is considered to be representative of siliceous plants^8^. At present, the available silicon content in paddy field soil can no longer meet the growth needs of rice, and the application of silicon fertilizer has become an important way to ensure rice yields^9^. Studies have shown that silicon application can promote rice root development and nutrient uptake by increasing the soil nutrient content^10^. The exogenous application of silicon-containing substances can improve rice photosynthesis, increase the ability of rice to resist drought, pests and diseases, and also effectively inhibit the absorption of heavy metals by rice^11^. For example, silicon can reduce the toxicity of Cd by adsorbing ions, regulating the activity of heavy metal transport proteases, and improving the antioxidant capacity^12,13^. Calcium is one of the essential elements for plant growth. Applying calcium-containing substances can improve the stability and integrity of plant cell membranes, promote plant root elongation and improve lodging resistance^14^. Studies have shown that exogenous application of calcium can promote the growth of rice, increase the antioxidant capacity of plants, and reduce the availability of heavy metals in the soil, thereby reducing the toxic effect of heavy metals in rice^15^. At the same time, calcium is an essential element for the human body. Applying calcium-containing substances can increase the calcium content in plants, thereby improving the calcium provision to the human body^16^. Silicon-calcium complex fertilizers are fertilizers mixed with silicates and carbonates, which can supply the silicon and calcium needed by rice. Research showed that silicon-calcium fertilizer can improve the structure and texture of rice soil, increase soil fertility, thereby strengthening the development and absorptive capacity of rice roots, improving rice’s resistance to diseases and pests and stress tolerance, and providing a more ideal growth environment for rice^17^.

At present, screening suitable soil passivation agents can maximize the efficiency of soil restoration, providing low-cost, environmentally friendly technology that considers green and sustainable development. Most of the research on soil remediation treatment focuses only on the change in soil properties or on the change of heavy metal content in rice. Less attention has been paid to comparing the effects of various soil amendments on rice quality, especially the impact on rice quality from the perspective of the soil environment. The factors affecting the field environment are relatively complex, and field experiments are more conducive to verifying the effect of amendments. Therefore, in this study, two early and two late rice varieties were selected, and three soil amendments were used to study their effects on physical and chemical soil properties, rice yield and quality in lightly cadmium-polluted paddy fields. Through a comprehensive analysis of the remediation effects, the study aimed to provide a scientific basis for the application of soil amendments in mildly cadmium-contaminated farmland and to ensure the safe production of rice.

## Materials and methods

### Test site and materials

The experiment was performed in a mildly Cd-contaminated paddy field in Hengyang County (26.970° N, 111.370° E), Hunan Province, China in March to October 2021. A typical double-cropping rice-growing system is used in the study area, which is in the central continental part of China. The site has a subtropical monsoon climate with annual precipitation of 1452 mm and a mean annual temperature of 17.9 °C. The mean Cd content of the soil at the study site was 0.47 mg kg^−1^, and the mean soil pH was 6.02. According to the Chinese National Soil Pollution Evaluation Technical Regulations, the soil was classed as mildly contaminated with Cd (Cd content 0.3–0.6 mg kg^−1^). The study site had red soil with a bioavailable Cd content of 0.12 mg kg^−1^, a cation exchange capacity of 7.57 cmol kg^−1^, an alkaline hydrolysable-N content of 119.88 mg kg^−1^, an available phosphorus content of 8.54 mg kg^−1^, an available potassium content of 57 mg kg^−1^, an organic matter content of 29.49 g kg^−1^, a total nitrogen content of 3.51 g kg^−1^, a total phosphorus content of 0.91 g kg^−1^, and a total potassium content of 10.13 g kg^−1^.

The early rice cultivars tested in the experiment were hybrid rice Zhuliangyou 819 and Luliangyou 996; the late rice cultivars were conventional rice Xiangwanxian 13 and Yuzhenxiang. Three soil amendments were selected for this study. Lime (CaO content ≥ 35% and MgO content ≥ 5%) was obtained from Hengyang Fulong Fertilizer Company (Hengyang, China) and had a Cd content of 0.15 mg kg^−1^. Biochar (produced by pyrolyzing rice husks at 550–600 °C for 2 h) was purchased from Wangcheng (Changsha, China) and had a Cd content of 0.18 mg kg^−1^. Silicon–calcium fertilizer (CaO content ≥ 25% and SiO_2_ content ≥ 20%) was obtained from Hunan Runbang Bioengineering Company (Changsha, China) and had a Cd content of 0.10 mg kg^−1^.

### Experimental design

A flat plot with uniform fertility was used for the experiment. Four treatments—blank control (CK treatment), biochar application at 10 t ha^−1^ (BC treatment), lime application at 1.5 t ha^−1^ (LM treatment), and SC application at 2.25 t ha^−1^ (SC treatment)—were set up (Table 1). All soil amendment was applied one week before the early rice seedlings were transplanted, and was not applied in the late rice. The soil amendment was spread evenly on the soil surface and the top soil was plowed to evenly mix the amendment and the soil.

**Table 1:** Trial treatment and specific measures.

Treatment	Code	Application method and dosage
Control	CK	Do not apply soil amendment, and fertilize according to local standards
Lime	LM	Lime was applied at an amount of 1.5 t ha^−1^, equivalent to 3.15 kg of per plot. Other indicators are the same as CK
Silicon–calcium fertilizer	SC	The silicon–calcium fertilizer was applied at an amount of 2.25 t ha^−1^, equivalent to 4.73 kg of per plot. Other indicators are the same as CK
Biochar	BC	Biochar was applied at an amount of 10 t ha^−1^, equivalent to 21 kg of per plot. Other indicators are the same as CK

This experiment used a two factor randomized block experiment with 4 treatments (three soil amendments and a control) and 2 varieties. Each treatment was repeated three times, and a 1.5 m-wide protective row was set up around it. Two varieties were used for early rice and late rice respectively, thus 24 plots, each 21 m^2^, were established. The surrounding area was separated using soil ridge of 0.3 m wide and 0.3 m high covered with polyethylene film. Independent irrigation and drainage outlets were installed. Water management was the same for each plot to exclude the effects of water on the Cd content of the soil. The field irrigation regimes in this experiment is intermittent irrigation (irrigating a 3 cm water layer each time, allowing it to dry naturally until there is no obvious water layer on the soil surface, and then filling a 3 cm water layer again, and this cycle continues until the rice maturity stage). Early rice was sown on 19 March and transplanted on 20 April, and late rice was sown on 21 June and transplanted on 21 July. The early rice transplanting density was 16.7 cm × 20 cm, the late rice transplanting density was 20 cm × 20 cm, and 2–3 seedlings were planted in each hole. Before rice transplanting, applied 600 kg ha^−1^ of compound fertilizer (N, P_2_O_5_, K_2_O ratio is 15:15:15), and applied 150 kg ha^−1^ of urea (nitrogen content 46.4%) at the peak tillering stage of rice. Other management practices were consistent with conventional local practices.

### Measurement items and methods

After the rice matured, 80 rice plants were randomly harvested in each plot, avoiding the three rows on the edge. After threshing, the straw and empty grains were removed and the grain was weighed. The moisture content was measured by the drying method, which was converted to the actual yield with a moisture content of 13.5%. The calculation formula was: actual yield = actual harvested yield × (1 − measured moisture content)/0.865. Then store the grain at room temperature for 3 months before conducting rice quality analysis.

Each sample was dehulled with a brown rice machine, and the brown rice rate of the sample was analyzed. A part of each brown rice sample was crushed with a stainless steel plant sample grinder, and the nitric acid-perchloric acid high-temperature digestion method was used to detect the Cd content in the digestive juice with an atomic absorption spectrophotometer. Another part of each brown rice sample were selected using the NP-4350 air separator, and the brown rice rate, milled rice rate and head milled rice rate were measured according to GB/T 17891-1999 “High Quality Rice”. Then, Another part of each milled rice sample were selected using the instrument JMWT12, and the length and width, chalkiness rate and chalkiness degree were tested according to GB/T 1354-2022 “Rice” japonica rice standard. Finally, the milled rice sample was ground into rice flour to determine the gel consistency, amylose content and protein content of the sample according to the “Rice Quality Determination Standard”^4^. The characteristic value analysis of the rice starch spectrum was measured with a fast viscometer produced by Australia Instrument Company and analyzed by TWC (Thermal Cycle for Win-dows) supporting software.

Samples of soil 0–20 cm deep were collected using a five-point sampling method before the soil amendment was added and when the early and late rice had matured. The soil samples were air dried, ground and passed through 20- and 100-mesh sieves. The soil pH was determined by extracting a sample with CO_2_-free distilled water at a water:soil ratio of 2.5:1 and then determining the pH using a PHSJ-3FX pH meter. For total Cd, 0.5 g of dry soil samples were weighed and digested with a mixed acid solution (HF-HClO_4_-HNO_3_) in a graphite digestion box (DS-360; China National Analytical Center, Guangzhou, China). For the bioavailable Cd, 5 g of dry soil sample with 0.1 mol CaCl_2_ solution (soil-to-liquid ratio 1:10) at 25 °C for 2 h at 250 rpm. Total Cd and CaCl_2_-extracted Cd concentrations were determined by graphite furnace atomic absorption spectrometry (AA800; Perkin Elmer, USA). The soil properties were determined according to Lin et al.^13^. The soil organic matter (SOM) was obtained by multiplying organic carbon with 1.724. Available N was determined by the alkaline hydrolysis diffusion method. Available phosphorus content was determined by colorimetric determination. Available potassium content was determined by flame photometer. Soil total nitrogen, total phosphorus and total potassium were determined by semi-micro KJELDAHL method.

### Operation formula

The formula for calculating the economic benefit is as follows:

$$ {\text{Increased production}}\,\,({\text{t ha}}^{{ - {1}}} )\, = \,{\text{Annual yeild}}\,\,({\text{soil amendment treatment}}) - {\text{Annual yeild}}\,\,({\text{CK treatment}}); $$

$$ {\text{Benefit}}({\text{dollar ha}}^{{ - {1}}} ) = {\text{Incremental production value}} - {\text{Cost}}. $$

### Statistical analysis

All statistical analyzes were completed using SPSS 22.0 software, and the LSD test was used to test for significant differences between treatments (p < 0.05). Origin 2021 draws graphics.

### Declaration

The manuscript file confirming that the experimental research and field studies on plants complied with relevant institutional, national, and international guidelines and legislation.

## Results

### Effects of different soil amendments on rice quality

#### Processing quality

Table 2 shows the effects of the soil amendments on the processing quality of different varieties of rice. Compared with the CK treatment, the BC and SC treatments significantly increased the rate of brown rice, milled rice and head milled rice (p < 0.05), while the LM treatment had no significant effect. For early rice, the rate of brown rice, milled rice and head milled rice increased by 9.60–12.71%, 8.16–8.96% and 8.45–14.01%, respectively, in the BC treatment compared with the CK treatment, and those of the SC treatment increased by 7.38–10.53%, 6.77–7.80% and 6.24–12.94%, respectively. For late rice, the rate of brown rice, milled rice and head milled rice increased by 8.44–8.60%, 7.66–11.05% and 11.89–13.50%, respectively, in the BC treatment compared with the CK treatment, and those of the SC treatment increased by 7.50–8.25%, 4.31–10.52% and 7.97–10.43%, respectively.

**Table 2:** Effects of soil amendments on rice processing and appearance quality.

Season	Cultivar	Treatment	BR (%)	MR (%)	HMR (%)	CR (%)	CD (%)	L/W (%)
Early rice	LLY-996	CK	72.56b	63.74b	55.97b	20.07a	4.67a	2.48a
		LM	75.46b	65.18ab	56.09b	16.90ab	4.28a	2.25a
		SC	80.20a	68.71a	59.46a	14.11b	3.33b	2.44a
		BC	81.78a	69.45a	60.70a	13.23b	2.92b	2.16a
	ZLY-819	CK	74.15b	62.60b	53.11b	18.96a	5.52a	3.20a
		LM	74.81b	61.28b	54.04b	16.56a	5.14a	3.05a
		SC	79.62a	66.84a	59.98a	11.14b	3.89b	3.26a
		BC	81.27a	67.71a	60.55a	10.91b	3.33b	2.95a
Late rice	YZX	CK	72.65b	59.91b	41.72b	15.19a	3.36a	4.41a
		LM	75.52ab	61.91b	44.98ab	12.70a	2.85a	4.37a
		SC	78.64a	66.21a	46.07a	7.14b	2.08b	4.35a
		BC	78.90a	66.53a	46.68a	6.43b	1.93b	4.23a
	XWX-13	CK	75.03b	60.04b	50.96b	14.25a	3.89a	2.59a
		LM	77.73b	60.41b	51.56b	11.29ab	3.54a	2.36a
		SC	80.66a	62.63ab	55.02a	8.92b	2.46b	2.86a
		BC	81.36a	64.64a	57.84a	7.36b	2.17b	2.65a

The different letters indicate that significant differences were found between the treatments for each cultivar using least significant difference tests.

*CK* control, *BC* biochar, *LM*, lime, *SC* silicon-calcium fertilizer, *BR* brown rice rate, *MR* milled rice rate, *HMR* head milled rice rate, *L/W* length/width ratio, *CR* chalkiness rate, *CD* chalkiness degree, *ZLY 819* Zhuliangyou 819, *LLY 996* Luliangyou 996, *YZX* Yuzhenxiang, *XWX 13* Xiangwanxian 13.

Values followed by different lowercase letters in the same group indicate significant difference among treatments n = 3, p < 0.05.

#### Appearance quality

Soil amendments had a significant effect on the appearance quality of rice, although this varied with different rice types. As shown in Table 2, compared with the CK treatment, there was no significant difference in the appearance quality of rice under the LM treatment of each cultivar, while the BC and SC treatments significantly reduced the chalkiness rate and chalkiness degree of rice (p < 0.05). For early rice, the chalkiness rate and chalkiness degree of rice decreased by 34.01–42.46% and 37.47–39.67%, respectively, in the BC treatment compared with the CK treatment, and those of the SC treatment increased by 29.70–41.24% and 28.69–29.53%, respectively. For late rice, the chalkiness rate and chalkiness degree of rice decreased by 48.35–57.67% and 42.56–44.22%, respectively, in the BC treatment compared with the CK treatment, and those of the SC treatment increased by 37.40–52.99% and 36.76–38.10%, respectively. Comparing the rice cultivars, the chalkiness rate and chalkiness degree of late conventional rice cultivars were lower than early hybrid rice cultivars.

#### Cooking quality

The RVA profile characteristics of rice treatment with different soil amendments are shown in Table 3. Except for the gelatinization temperature and peak time, the other indicators of the RVA profile were significantly different among different soil amendment treatments and cultivar types. Compared with the CK treatment, the BC and SC treatments significantly decreased the peak viscosity, hot pulp viscosity, final viscosity and setback of rice, and significantly increased the breakdown (p < 0.05), while the LM treatment made no significant difference. A high peak and trough viscosity, large breakdown value, and small setback value lead to high eating value. Therefore, the results of comparison among different cultivars showed that the characteristic of the RVA profile of late conventional rice was better than that of early hybrid rice, which was consistent with the results of amylose content and gel consistency.

**Table 3:** Effects of soil amendments on rice rapid viscosity analyzer (RVA) profile characteristics.

Season	Cultivar	Treatment	PV (cp)	HV (cp)	FV (cp)	BD (cp)	SB (cp)	PAT (°C)	PET (min)
Early rice	LLY-996	CK	4755a	2290a	3259a	1434a	206b	75.20a	5.67a
		LM	4710a	2250a	3126ab	1422a	237b	75.20a	5.67a
		SC	4367b	2012b	3061b	1268b	308a	75.55a	5.63a
		BC	4353b	2089b	3054b	1332b	345a	76.25a	5.67a
	ZLY-819	CK	4666a	2501a	3481a	2315a	− 454c	73.65b	5.73a
		LM	4531a	2330ab	3388ab	2193a	− 365b	73.35b	5.87a
		SC	4327b	2138b	3266b	1800b	− 332b	75.65a	5.73a
		BC	4259b	2188b	3239b	1702b	− 255a	75.55a	5.80a
Late rice	YZX	CK	5338a	3932a	5539a	2675a	− 1729b	78.93b	5.88a
		LM	5221a	3850a	5428a	2620a	− 1701b	79.35b	5.93a
		SC	5108b	3774ab	5226b	2438b	− 1606ab	82.60a	5.87a
		BC	5054b	3687b	5199b	2441b	− 1599a	83.55a	6.00a
	XWX-13	CK	5444a	3351a	4790a	2428a	− 1371b	82.00a	5.80a
		LM	5366a	3227a	4701a	2430a	− 1350b	82.30a	5.67a
		SC	5055b	2966b	4187b	2339ab	− 1188a	84.00a	5.80a
		BC	5042b	2853b	4087b	2229b	− 1100a	84.45a	5.47a

The different letters indicate that significant differences were found between the treatments for each cultivar using least significant difference tests.

*CK* control, *BC* biochar, *LM* lime, *SC* silicon–calcium fertilizer, *PV* Peak viscosity, *HV* Hot paste viscosity, *FV* Final viscosity, *BD* Breakdown, *SB* Setback, *PAT* Pasting temperature, *PET* Peak time, *ZLY 819* Zhuliangyou 819, *LLY 996* Luliangyou 996, *YZX* Yuzhenxiang, *XWX 13* Xiangwanxian 13.

Values followed by different lowercase letters in the same group indicate significant difference among treatments n = 3, p < 0.05.

The amylose content and gel consistency are important indicators for evaluating the taste of rice. As shown in Table 4, there was no significant difference in the amylose content of early and late rice cultivars under different treatments, but there were differences among cultivars. The amylose content of the late rice cultivars (YZX and XWX-13) was generally lower than that of the early rice cultivars (LLY-996 and ZLY-819). Compared with the CK treatment, there was no significant difference in the gel consistency of the LM treatment, but the BC and SC treatments significantly increased the gel consistency of rice (p < 0.05). For early rice, the gel consistency of the BC treatment increased by 17.09–31.43% compared with the CK treatment, and that of the SC treatment increased by 11.97–32.86%. For late rice, the gel consistency of the BC treatment increased by 13.14–17.91% compared with the CK treatment, and that of the SC treatment increased by 10.22–13.43% compared with the CK treatment.

**Table 4:** Effects of soil amendments on the cooking, nutrition and hygiene quality of rice.

Season	Cultivar	Treatment	AC (%)	GC (mm)	PC	BR-Cd (mg kg^−1^)
Early rice	LLY-996	CK	26.77a	35.0b	7.09b	0.206a
		LM	26.93a	39.5b	7.61b	0.146b
		SC	26.72a	46.5a	8.14a	0.140b
		BC	27.15a	46.0a	8.84a	0.154b
	ZLY-819	CK	22.96a	58.5b	6.21b	0.155a
		LM	22.45a	61.5b	6.94b	0.113b
		SC	22.32a	65.5a	8.94a	0.108b
		BC	22.49a	68.5a	9.47a	0.131ab
Late rice	YZX	CK	15.79a	67.0b	6.90b	0.197a
		LM	15.57a	70.5b	7.13b	0.119b
		SC	15.36a	76.0a	7.92a	0.116b
		BC	15.40a	79.0a	8.35a	0.132b
	XWX-13	CK	14.67a	68.5b	6.39b	0.128a
		LM	14.80a	71.0b	7.37ab	0.094b
		SC	14.59a	75.5a	7.76a	0.091b
		BC	14.72a	77.5a	7.99a	0.101b

The different letters indicate that significant differences were found between the treatments for each cultivar using least significant difference tests.

*CK* control, *BC* biochar, *LM* lime, *SC* silicon–calcium fertilizer, *AC* amylose content, *GC* gel consistency, *PC* protein content, *BR-Cd* brown rice Cd content, *ZLY 819* Zhuliangyou 819, *LLY 996* Luliangyou 996, *YZX* Yuzhenxiang, *XWX 13* Xiangwanxian 13.

Values followed by different lowercase letters in the same group indicate significant difference among treatments n = 3, p < 0.05.

#### Nutrition quality

The protein in milled rice is more easily digested and absorbed by the human body than the protein in any other grains, thus milled rice has been identified as an excellent vegetable protein. As shown in Table 4, compared with the CK treatment, the BC and SC treatments significantly increased the rice protein content (p < 0.05), while the LM treatment showed no change. For early rice, the protein content of the BC treatment significantly increased by 24.68–52.49% compared with the CK treatment, and that of SC treatment significantly increased by 14.81–43.96% (p < 0.05). For late rice, the protein content of the BC treatment significantly increased by 21.01–25.04% compared with the CK treatment, and that of the SC treatment was significantly increased by 14.78–21.43% (p < 0.05).

#### Hygiene quality

As shown in Table 4, all three soil amendments could significantly reduce the Cd content in rice (p < 0.05), and the Cd reduction effects among the soil amendments were consistent. Compared with the CK treatment, the soil amendments significantly reduced the rice Cd content of LLY-996 rice by 25.24–32.04%, significantly reduced the rice Cd content of ZLY-819 rice by 15.48–30.32%, significantly reduced the rice Cd content of YZX by 32.99–41.17%, and significantly reduced the rice Cd content of XWX-13 by 21.09–28.91% (p < 0.05). Comparatively speaking, the Cd reduction effect of SC was the greatest, followed by LM and then BC.

### Effects of different soil amendments on soil properties

**Figure 1:**
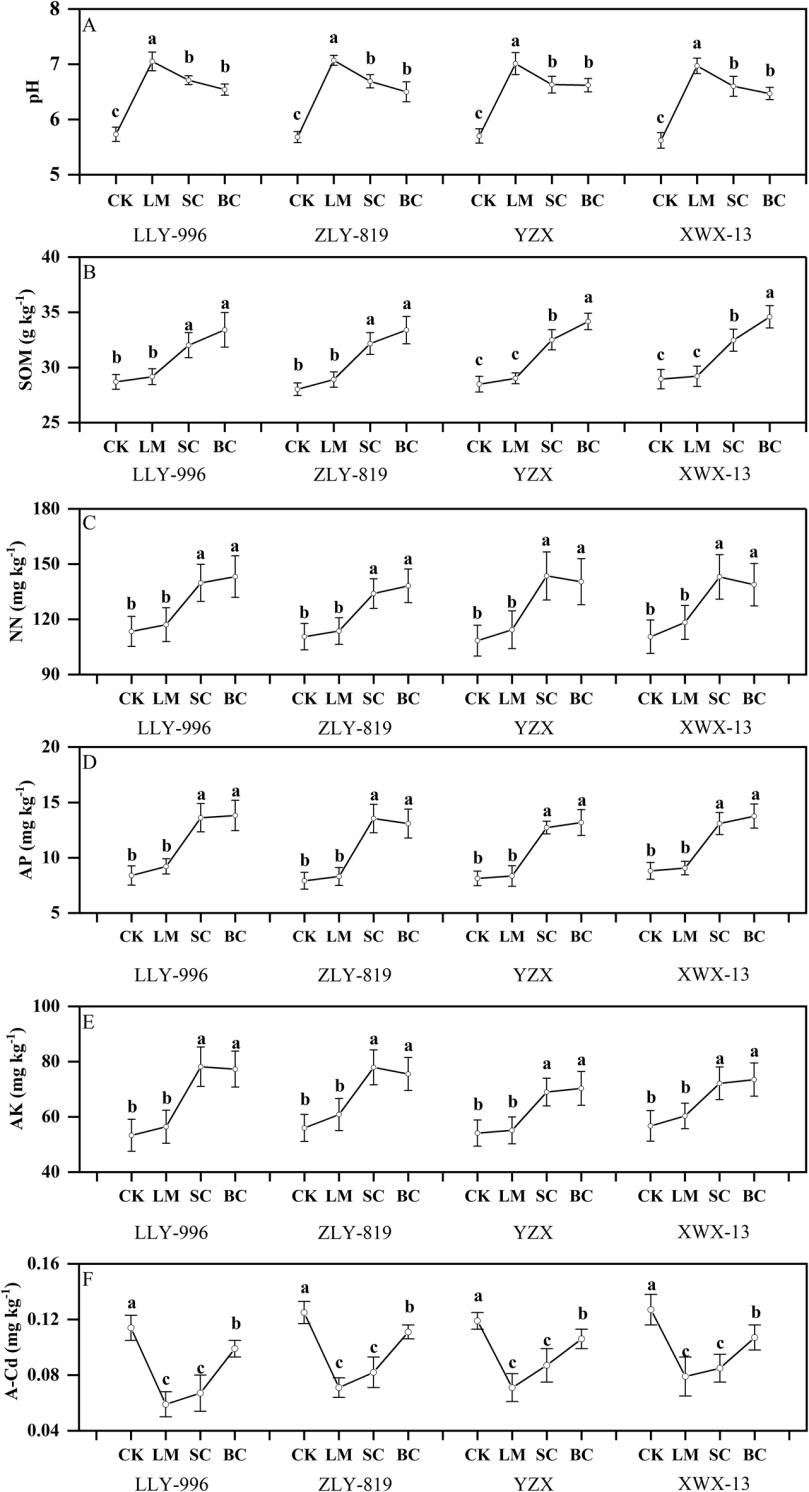
Effect of soil amendment on soil properties. *CK* control, *BC* biochar, *LM* lime, *SC* silicon–calcium fertilizer. pH (**A**); *SOM* soil organic matter (**B**); *NN* alkaline hydrolysable-N (**C**); *AP* available phosphorus (**D**); *AK* available potassium (**E**); *A-Cd* available cadmium (**F**). *ZLY 819* Zhuliangyou 819, *LLY 996* Luliangyou 996, *YZX* Yuzhenxiang, *XWX 13* Xiangwanxian 13. The different letters indicate that significant differences were found between the treatments for each cultivar using least significant difference tests. The numeric values represent mean ± standard error. Values followed by different lowercase letters in the same group indicate significant difference among treatments n = 3, p < 0.05.

The application of the soil amendments had different effects on soil physical and chemical indicators. As shown in Fig. 1, the application of soil amendments could significantly increase soil pH (A), and the effect was LM > SC > BC (p < 0.05). The soil pH of the LM treatment was significantly higher than that of the SC and BC treatments. On the basis of the results of the four cultivars, compared with CK, the soil pH of LM, SC, and BC increased by 1.31–1.39, 0.93–1.01, and 0.81–0.92 unit, respectively.

From the perspective of soil SOM (B) change, the LM treatment had no significant effect on soil organic matter, while the BC and SC treatments significantly increased soil organic matter. For early rice, compared with CK, the SC and BC treatments significantly increased soil organic matter by 11.57–14.77% and 16.41–18.91%, respectively. For late rice, compared with CK, the SC and BC treatments significantly increased soil organic matter by 12.16–13.91% and 19.48–19.94%, respectively (p < 0.05).

The soil NN (C), AP (D) and AK (E) under the SC and BC treatments all had an increasing trend compared with the CK treatment, and the effects of the two soil amendments were consistent (Fig. 1). For early rice, compared with CK, the NN, AP and AP in the SC treatment increased significantly by 21.17–23.22%, 62.34–71.09% and 39.20–46.57%, and those of the BC treatment increased significantly by 24.96–26.25%, 64.84–65.28% and 34.95–44.94%, respectively (p < 0.05). For late rice, compared with CK, the NN, AP and AP in the SC treatment increased significantly by 29.39–32.47%, 48.58–56.58% and 27.21–27.47%, while those of the BC treatment increased significantly by 25.56–29.57%, 56.30–62.24% and 29.59–29.98%, respectively (p < 0.05).

The three soil amendments had a significant effect on reducing the soil A-Cd (F). Consistent with the pH results, the degree of reduction was LM > SC > BC, and the reductions with the LM and SC treatments were significantly higher than those with the BC treatment. Compared with CK, the Cd content in LM, SC, and BC soils decreased significantly by 37.79–48.24%, 26.89–41.23% and 10.92–15.74%, respectively (p < 0.05).

### Correlation analysis of soil properties and rice quality

**Figure 2:**
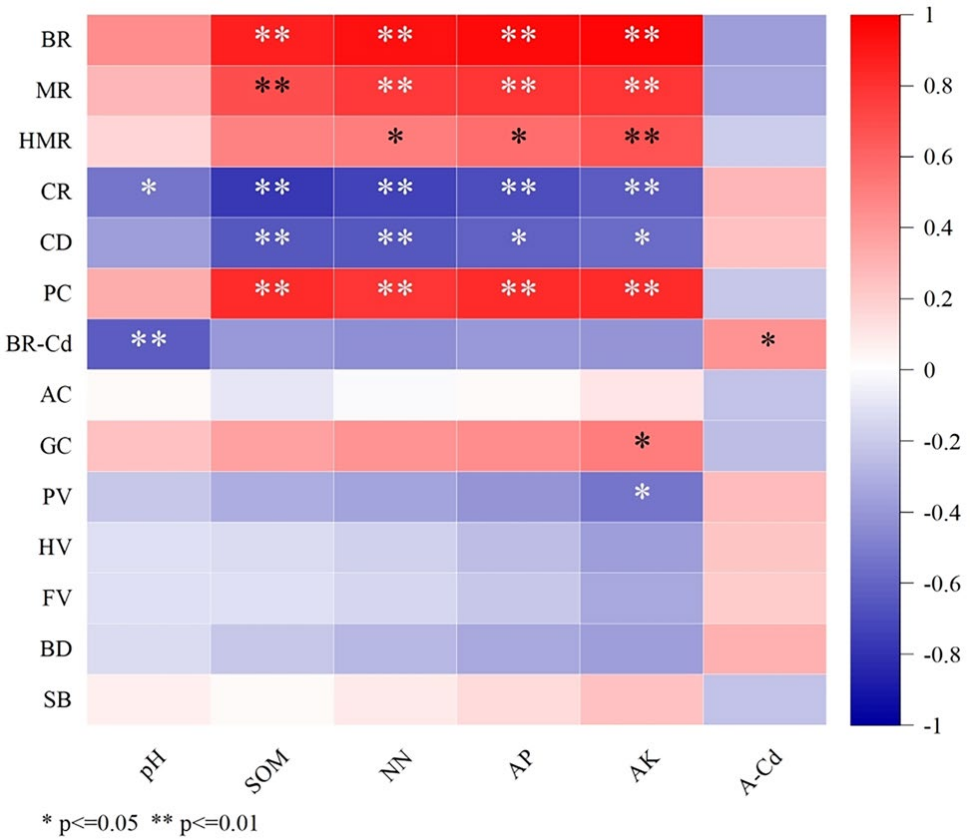
Correlation analysis between soil properties and rice quality. *Significant at the 0.05 level, **Significant at the 0.01 level. *SOM* soil organic matter, *NN* alkaline hydrolysable-N, *AP* available phosphorus, *AK* available potassium, *A-Cd* available cadmium, *BR* brown rice rate, *MR* milled rice rate, *HMR* head milled rice rate, *CR* chalkiness rate, *CD* chalkiness degree, *PC* protein content, *BR-Cd* brown rice Cd content, *AC* amylose content, *GC* gel consistency, *PV* peak viscosity, *HV* hot paste viscosity, *FV* final viscosity, *BD* breakdown, *SB* setback.

The correlation analysis of rice processing, appearance, cooking, nutrition and hygiene quality and soil properties is shown in Fig. 2. The brown rice rate and milled rice rate in rice processing quality were significantly positively correlated with SOM, NN, AP and AK (brown rice rate: R = 0.87–0.97, p < 0.01; milled rice rate R = 0.70–0.80, p < 0.01). The head milled rice rate was significantly positively correlated with soil available nutrients (R = 0.52–0.67, p < 0.05). The chalkiness rate and chalkiness degree of rice appearance quality were significantly or extremely significantly negatively correlated with pH, SOM and available nutrients (chalkiness rate: R = − 0.54–0.78, p < 0.01; chalkiness degree: R =  − 0.56–0.64, p < 0.05). A significant positive correlation existed between gel consistency and AK (R = 0.51, p < 0.05). The amylose content and RVA profile characteristics were not significantly correlated with soil properties indicators. Protein content had an extremely significant positive correlation with SOM and available nutrients (R = 0.79–0.84, p < 0.01). The rice Cd content in rice hygienic quality was significantly negatively correlated with pH (R =  − 0.63, p < 0.01), and significantly positively correlated with A-Cd (R = 0.50, p < 0.05).

### Effects of different soil amendments on rice yield and economic benefit

**Figure 3:**
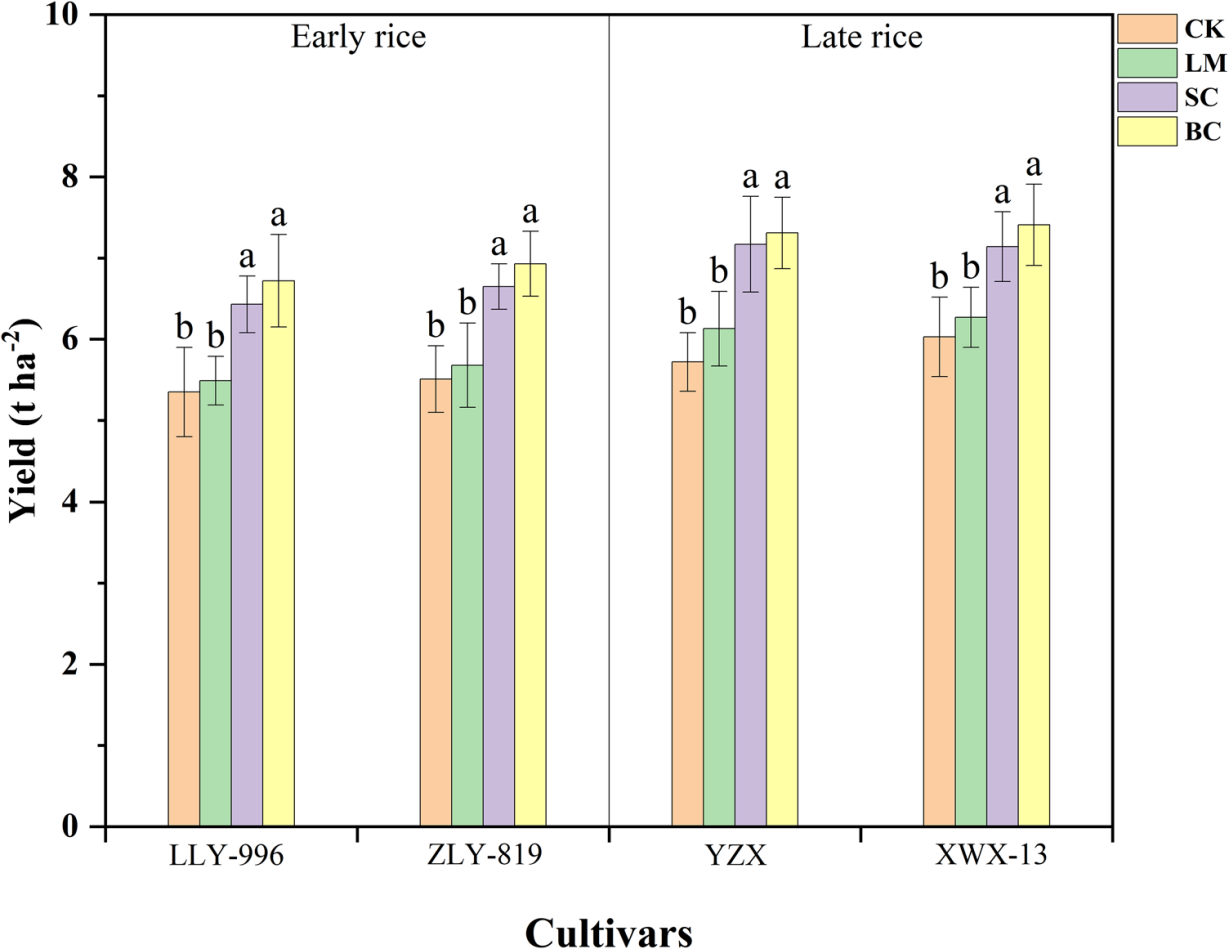
Effect of soil amendment on rice yield. *CK* control, *BC* biochar, *LM* lime, *SC* silicon–calcium fertilizer, *ZLY 819* Zhuliangyou 819, *LLY 996* Luliangyou 996, *YZX* Yuzhenxiang, *XWX 13* Xiangwanxian 13. The different letters indicate that significant differences were found between the treatments for each cultivar using least significant difference tests. The numeric values represent mean ± standard error. Values followed by different lowercase letters in the same group indicate significant difference among treatments n = 3, p < 0.05.

As shown in Fig. 3 and Table 5, the application of soil amendments could increase the rice yield. The yield of each cultivar was in the order of BC > SC > LM > CK, and the BC and SC treatments were significantly higher than the CK treatment (p < 0.05). The average early rice yields for the BC and SC treatments were 6.83 t ha^−1^ and 6.54 t ha^−1^, which was an increase of 25.78% and 20.44% compared with the CK treatment. The average yields of late rice were 7.35 t ha^−1^ and 7.13 t ha^−1^, which was an increase of 24.93% and 21.25%, respectively, compared with the CK treatment. It can be seen that the application of biochar and silicon-calcium fertilizer can significantly increase rice yield.

**Table 5:** Economic benefit analysis with different soil amendments.

Treatments	Annual yield (t ha^−1^)	Increased production (t ha^−1^)	Incremental production value (dollar ha^−1^)	Cost (dollar ha^−1^)	Benefit (dollar ha^−1^)
CK	11.31	–	–	–	–
LM	11.79	0.48	192	87.72	104.28
SC	13.70	2.39	956	394.74	561.26
BC	14.29	2.98	1192	2339.18	− 1147.18

The price of rice is 400 US dollars t^−1^ (RMB to US dollar exchange rate 1:6.84).

*CK* control, *BC* biochar, *LM* lime, *SC* silicon–calcium fertilizer.

Table 5 shows a trend of BC > SC > LM in terms of the effect of soil amendments on increased rice yield. However, in terms of benefit analysis combined with cost, the BC treatment showed a negative benefit effect, while the LM treatment and the SC treatment showed a positive benefit effect. The SC treatment had the highest benefit effect.

## Discussion

### Effects of soil amendments on soil properties

In the current study, the soil amendments significantly increased soil pH and reduced soil available Cd content, among which lime was more effective than biochar and silicon–calcium fertilizer. Beacuse the application of lime is mainly aimed at increasing the soil pH value, thereby increasing the soil redox potential and reducing the availability of heavy metals in the soil^18^. However, lime application had no significant effect on the soil’s available nutrient content (SOM, NN, AP, AK). In contrast, the BC treatment with biochar application and SC treatment with silicon–calcium fertilizer significantly increased the soil nutrient content. As an alkaline compound fertilizer, biochar can promote the growth of crops by improving the physical and chemical environment of the soil. Applying biochar to the soil can increase the pH value of the acidified soil, reduce the loss of soil nitrogen and effectively alleviate the release of fertilizers in the soil, thus meeting the needs of crops for nutrients in the later stages of growth^19^. According to reports, the application of silicon fertilizer can regulate the pH and alleviate soil acidification, and it also contains some nutrients, which have the effect of fertilizing the soil^20^. Wang et al.^21^ reported that the application of multi-element mineral soil amendments can increase the diversity of soil microorganisms, especially the richness of species, enhance the activity of soil urease, catalase and other enzymes, introduce organic carbon sources into the soil, and directly increase the content of soil organic matter. The increase effect of the BC treatment in late rice was significantly higher than that of the SC treatment, which may have been due to the more thorough decomposition of biochar by microorganisms over time, which was more conducive to the accumulation of nutrients in the soil^22^.

### Effect of soil amendments on rice quality

The formation process of rice quality is essentially the carbon and nitrogen metabolism in rice and the process of grain filling^23^. In addition to the genetic characteristics of cultivars, environmental conditions and cultivation measures, especially artificial application of soil amendments, are important factors affecting rice quality^24^. The application of organic carbon can reduce the soil bulk density and promote the nutrient absorption of rice roots, while the nutrients produced after its decomposition can increase the nitrogen content in the plant and the synthesis of related enzymes and improve the photosynthesis of leaves. This promotes the transfer of nutrients from all parts of the plant to the ear, which increases the hardness of the grain, enhances the ability of rice to resist milling damage and reduces the formation of rice chalkiness^25^. A previous study on the effect of lime on rice did not find that it had a significant effect on the rice quality indicators, although the application of CaO did significantly reduce the Cd content of brown rice^26^. However, in tomato research, it was found that the application of calcium fertilizer soil amendment could improve tomato quality, and the content of soluble solids increased by 20.2%^27^. Studies have reported that silicon–calcium fertilizer can enhance the activity of glutamic acid aminotransferase in rice leaves, increase the chlorophyll content and photosynthetic rate of leaves and, thus, increase the accumulation of photosynthetic substances after heading^21,28^.

In this study, the application of biochar and silicon-calcium fertilizer increased the brown rice rate, milled rice rate and head milled rice rate, and reduced the chalkiness rate and chalkiness degree of rice, and the two effects were relatively consistent. These findings indicated that the application of biochar and silicon-calcium fertilizer could increase the hardness of rice, reduce the occurrence of chalkiness, and improve the processing quality and appearance quality of rice. Studies have shown that the dominant factor affecting rice processing quality and appearance quality is grain filling. When the grain filling is fuller, the endosperm cells and tissues are more abundant and there are smaller internal gaps in the grain; when the arrangement is tighter, the hardness of the grain is higher and the probability of chalk formation is lower^29,30^. Both biochar and silicon-calcium fertilizer improve soil properties, increase the photosynthetic rate of rice leaves, prevent premature senescence of leaves, prolong the effective filling period of grains and enhance the transfer of filling materials to grains, thus increasing grain quality and fullness.

Rice with a low amylose content and long gel consistency has better cooking quality. Gel consistency is influenced by environment more than genotype, while amylose content shows the opposite influences^31^. In the results of the current study, the BC treatment and the SC treatment significantly improved rice gel consistency but had no significant effect on amylose content. It may be that both biochar and silicon-calcium fertilizer can promote the absorption of nutrients by rice by increasing the nutrient content of the soil, improve the photosynthesis of plant leaf, and promote the transport of nutrients into the grains, thereby increasing the gel consistency of rice^32,33^.

There was a significant correlation between RVA profile and cooking quality. Studies have shown that rice with high peak viscosity, large breakdown and small setback value was considered to have better cooking quality^34^. Protein content is not only the key factor affecting the cooking quality, but also the main index to measure the nutritional quality of rice. Studies have shown that the protein content is negatively correlated with the cooking quality of rice but positively correlated with the nutritional quality^35^.

The results of the current study showed that, for the RVA profile characteristics, the BC treatment and the SC treatment significantly reduced the peak viscosity, hot pulp viscosity, final viscosity and breakdown of rice, while significantly they increased the setback. The protein content of rice treated with BC and SC increased significantly by 21.01–52.49% and 14.78–43.96%, respectively, compared with the CK treatment (p < 0.05). Thus, the application of biochar and silicon-calcium fertilizer can improve the nutritional quality of rice to a certain extent, but at the same time can reduce its eating quality. In this study, both biochar and silicon-calcium fertilizer could significantly increase the soil nutrient content, especially alkaline hydrolysable-N. This promotes the absorption of carbon and nitrogen in the soil by rice, improves the carbon and nitrogen assimilation ability of the plant, promotes the transport of assimilated substances in various vegetative organs to the grain, stimulates the activities of enzymes related to starch synthesis and nitrogen assimilation enzymes in the grain and increases protein synthesis^36^. Studies have shown that the protein content has a significant negative correlation with breakdown and peak viscosity, and a very significant positive correlation with setback^37^. The results of the current study are basically consistent with the previous ones. Because of the higher protein content, the pores between starch grains are reduced, and the structure of rice grains is denser, which leads to slower water absorption and insufficient gelatinization of starch, which eventually leads to reduced palatability^38^. Therefore, further study is needed into how to regulate the structure of protein components and their correlation with the nutritional and eating qualities of rice.

Soil amendments generally reduce Cd content in brown rice in several ways. The application of alkaline amendments increases the soil pH value, promotes the precipitation of Cd, and reduces the available Cd content in the soil, thereby reducing the absorption of Cd by rice^39^. In regulating soil acidification, lime and biochar are common amendments. Lime reduces the content of exchangeable Cd in the soil by increasing the cation exchange capacity in the soil. Biochar is a porous carbon structure, which can chelate Cd through abundant oxygen-containing functional groups (acidic functional groups such as carboxyl and phenolic hydroxyl groups) on the surface^40^. In the current study, the SC treatment had the best effect on reducing Cd content in brown rice. The composition of silicon–calcium fertilizer was: CaO ≥ 25%, SiO_2_ ≥ 20%, K_2_O + P_2_O_5_ ≥ 3%. The calcium element in its composition can increase the cation exchange capacity in the soil, and the silicon element can combine with the Cd in the soil to form a compound that cannot be absorbed by plants. At the same time, silicon can also improve the growth of rice, induce cells to produce small molecular chelating agents, enhance the chelation of Cd and reduce the content of free Cd in cells^41^.

### Correlation between soil properties and rice quality

Rice quality is mainly determined by the interaction between genotype and environment. Environmental influences have largely been considered as single factors interacting with specific genotypes of specific plants. Because plants grow in changing environments, plants at different growth stages are affected by changes in environmental conditions even when grown in the same area^42^. Therefore, it is necessary to investigate the impact of environmental factors on specific crops.

In the current study, soil nutrient indexes (SOM, NN, AP, AK) were significantly positively correlated with rice processing quality and nutritional quality, and significantly negatively correlated with appearance quality. They were not significantly correlated with cooking and eating quality and hygienic quality. Soil pH was significantly negatively correlated with brown rice Cd content, and soil available Cd content was significantly positively correlated with brown rice Cd content. The analysis showed that the application of soil amendments increased the soil nutrient content (SOM, NN, AP, AK), and the increase in soil nutrient content significantly improved the growth and development of rice, which in turn promoted the improvement of rice processing, appearance and nutritional quality. The effect of soil amendment on the hygienic quality of rice is mainly through increasing the soil pH and reducing the available Cd content in the soil, thereby reducing the Cd content in brown rice. In summary, the improvement in rice processing, appearance and nutritional quality was mainly affected by the comprehensive effects of available nitrogen, phosphorus, potassium and organic matter content in the soil. Hygiene quality was mainly affected by soil pH and available Cd content. Cooking and eating quality had little correlation with soil properties. Among the environmental factors, nitrogen is an important factor affecting rice quality and nitrogen is the main element in the synthesis of major nutrients such as protein and starch in rice grains. Therefore, increasing the available nitrogen content in the soil can promote the absorption of nitrogen elements by rice, increase the nitrogen content in various organs of the plant, and promote the transport of nitrogen elements into the grains, thereby improving the rice quality^43^. Studies have shown that the impact of potassium on rice quality is second only to nitrogen and increasing the soil potassium content can effectively improve the nutritional and eating quality of rice^44^. It has also been reported that the application of appropriate amounts of phosphorus can improve the aroma, taste and cooking and eating quality of rice. It has also been reported that spraying foliar phosphorus and silicon fertilizers can improve the aroma, taste, texture and cooking and eating quality of rice^45^.

### Effects of different soil amendments on rice yield and economic benefits

In this study, all three soil amendments could increase rice yield. However, the cost and potential risks of their application must be considered before these amendments can be applied to large-scale practical production fields. As shown in Table 5, among the soil amendments, only lime and silicon-calcium fertilizer could improve the economic benefits of rice planting. The SC treatment had the highest synergistic effect, reaching 561.26 US dollars ha^−1^, followed by LM, which was 104.28 US dollars ha^−1^. Although biochar can significantly improve rice yield and rice quality, the high cost has been an important reason hindering farmers from widely accepting it. In contrast, silicon-calcium fertilizers are low in cost and perform better in terms of high yield and high efficiency and they are worthy of large-scale promotion and application.

Additionally, limitations of this study include the fact that the experiment was conducted in one region for only 1 year. This experimental location was conducted on a lightly cadmium contaminated rice field and generalizability to other regions or soil types may be limited. Considering the effects of soil nutrient properties, temperature and light conditions on yield and quality of rice plants. This study may need to be strengthened through multiple years and multiple regions to verify the results of this study. At the same time, the physiological and molecular mechanisms underlying the observed differences will need to be clarified, which may lead to the development of methods for the safe and stable production of rice.

## Conclusions

In this study, the application of biochar and silicon–calcium fertilizer significantly improved rice processing, appearance and nutritional quality, and lime significantly improved rice hygienic quality. The application of lime and silicon–calcium fertilizer significantly increased pH and decreased A-Cd content. The application of biochar and silicon–calcium fertilizer significantly increased the content of SOM, NN, AP and AK. Correlation analysis found that biochar and silicon–calcium fertilizer increased the soil nutrient content (SOM, NN, AP, AK), and thus promoted the improvement of rice processing, appearance and nutritional quality. In terms of economic benefit, silicon–calcium fertilizer had the highest benefit in increasing income among the soil amendments. On the whole, in the mildly Cd-polluted rice fields, the application of silicon–calcium fertilizer had the best improvement effects on the soil quality, the yield and quality of rice, and the economic benefits.

## Data Availability

The data that support the findings of this study are available from the corresponding author upon reasonable request.
