# Author Correction: Silicon–calcium fertilizer increased rice yield and quality by improving soil health

**DOI:** 10.1038/s41598-024-69766-w

**Published:** 2024-08-28

**Authors:** Shuai Yuan, Yu Han, Can Cui, Pingping Chen, Naimei Tu, Zhongwen Rang, Zhenxie Yi

**Affiliations:** College of Agronomy, Hunan Agricultural University, Changsha, 410128 China

Correction to: *Scientific Report* 10.1038/s41598-024-63737-x, published online 07 June 2024

The Funding section in the original version of this Article omitted a project number. The Funding section,

This work was financially supported by the National Key R&D Program of China (2018YFD0301005, 2017YFD0301501), and the Hunan Provincial Department of Agricultural and Rural Affairs Project grant number “XIANG CAI JIAN ZHI” (2023, No. 98).

now reads,

This work was financially supported by the National Key R&D Program of China (2018YFD0301005, 2017YFD030150, 2023YFD2301400), and the Hunan Provincial Department of Agricultural and Rural Affairs Project grant number “XIANG CAI JIAN ZHI” (2023, No. 98).

The original Article has been corrected.

